# The Burt Case

**Published:** 1898-03

**Authors:** 


The Burt Case.—Eugene Burt, youngest son of the late Dr. Wm. Jefferson Burt, long time Secretary of the Texas State Medical Association, it will be remembered, was convicted at Austin in October last of the murder of his wife and two babies under circumstances of a most revolting nature. The plea of insanity was set up as a defense. Numerous physicians testified as experts, and he was pronounced "sane" and sentenced to be hanged at Austin on the 21st of January last. Every effort to get a reversal of the verdict failed, the Appellate Court affirming the finding of the trial court, and but one chance to save him from the gallows remained. His lawyers availed themselves of that one chance, and secured a stay of execution, within an hour of the time appointed for his execution.

He is to be tried for "present insanity," as the law cannot, or, rather should not hang an insane man. The law provides that if any reputable person will make an affidavit that a person under death sentence is insane, that person shall be tried for insanity, and if found to be insane he shall be put in the asylum, and if he ever becomes sane, then the sentence of the court shall be executed. Burt's brother made the affidavit, and the trial is set for the 31st of March, inst. The result of this trial will be watched with keen interest by all classes of society. A petition numerously signed, asking the Governor to commute the sentence to life imprisonment in the penitentiary was refused by Governor Culberson. A feeling of bitter animosity towards the unfortunate man exists in Austin, and it is thought by many that he had not, really, a fair trial. In the opinion of the writer he is undoubtedly insane, and has been for a number of years.

